# Rapid Male-Specific Regulatory Divergence and Down Regulation of Spermatogenesis Genes in Drosophila Species Hybrids

**DOI:** 10.1371/journal.pone.0061575

**Published:** 2013-04-11

**Authors:** Jennifer Ferguson, Suzanne Gomes, Alberto Civetta

**Affiliations:** Department of Biology, University of Winnipeg, Winnipeg, Manitoba, Canada; North Carolina State University, United States of America

## Abstract

In most crosses between closely related species of Drosophila, the male hybrids are sterile and show postmeiotic abnormalities. A series of gene expression studies using genomic approaches have found significant down regulation of postmeiotic spermatogenesis genes in sterile male hybrids. These results have led some to suggest a direct relationship between down regulation in gene expression and hybrid sterility. An alternative explanation to a cause-and-effect relationship between misregulation of gene expression and male sterility is rapid divergence of male sex regulatory elements leading to incompatible interactions in an interspecies hybrid genome. To test the effect of regulatory divergence in spermatogenesis gene expression, we isolated 35 fertile *D. simulans* strains with *D. mauritiana* introgressions in either the X, second or third chromosome. We analyzed gene expression in these fertile hybrid strains for a subset of spermatogenesis genes previously reported as significantly under expressed in sterile hybrids relative to *D. simulans*. We found that fertile autosomal introgressions can cause levels of gene down regulation similar to that of sterile hybrids. We also found that X chromosome heterospecific introgressions cause significantly less gene down regulation than autosomal introgressions. Our results provide evidence that rapid male sex gene regulatory divergence can explain misexpression of spermatogenesis genes in hybrids.

## Introduction

Sterility of the heterogametic sex is one of the most frequent results for hybrids produced from crosses between closely related species [1]. In species of the Drosophila genus, where the male is the heterogametic sex, the sterile hybrid males have mostly atrophied seminal vesicles with normal testes morphology, although a small proportion of hybrids might also show whole or partial testes atrophy [2]–[4]. The morphological anomaly of the seminal vesicle is indicative of late sperm developmental problems in hybrids, leading to reductions in, or nearly complete loss of sperm production. In fact, cytological studies have shown for the most part, that sterile hybrid males are able to proceed normally through sperm development until the meiotic division stage, but several postmeiotic problems are detected. These include lack of synchrony in spermatid development, failure to complete spermatid individualization, large amounts of cellular debris between each sperm bundle and formation of undifferentiated interconnected spermatids [5]–[7]. Sperm development abnormalities in interspecies sterile hybrids are not limited to Drosophila. Interspecies hybrids between species of *Xenopus* have a larger proportion of undifferentiated spermatids and more immotile sperm than parental species, while house mouse hybrids show a wide range of both meiotic and postmeiotic defects [8]–[10].

The process of spermatogenesis is well characterized in Drosophila. Germline stem cells divide into daughter cells that maintain the supply of germ cells, while others (spermatogonia) continue to divide mitotically, forming primary spermatocytes. The primary spermatocytes enter meiosis in synchrony to produce spermatid bundles which later mature into single sperm cells through the process of spermiogenesis [11]–[14]. Several genes have been identified that play different roles during sperm development, and while cellular and morphological events appear to be independently regulated, some genes act as key regulators of developmental transitions. For example, both *bag of marbles* (*bam*) and *benign gonial cell neoplasm* (*bgcn*) limit the number of mitotic divisions, allowing the transition from mitosis into meiosis, while *always early* (*aly*) and *cookie monster* (*comr*) are members of a class of genes known as meiotic arrest genes that are needed for the progression of meiosis and the start of spermiogenesis [12], [15], [16].

The fact that sperm cell development is disrupted in Drosophila interspecies sterile hybrids, combined with our knowledge of spermatogenesis gene function in *Drosophila melanogaster*, has recently led to a series of studies comparing patterns of spermatogenesis gene expression in fertile parental species and sterile hybrids. Despite some contradictory results between studies, more postmeiotic (spermiogenesis) than meiotic and premeiotic genes have been found to be significantly under expressed in sterile hybrids compared to parental species [17]–[22]. For example, a study that used a custom made species-specific sperm array found three and six spermatid differentiation genes down regulated in *D. simulans* - *D. mauritiana* and *D. simulans - D. sechellia* hybrids respectively, compared to a single meiotic arrest gene [19].

Transcription of genes of spermatogenesis is mostly premeiotic, with a few exceptions [23]–[25]. Thus, misexpression at the transcriptional level could not be a consequence of postmeiotic problems linked to sterility unless subtle unknown premeiotic problems have gone undetected in prior microscopy studies of spermatogenesis in sterile hybrids. Because transcripts accumulate premeiotically, it has been hypothesized that down regulation of postmeiotic spermatogenesis genes in sterile hybrids might be a causative factor of hybrid male sterility [18]. Alternatively, down regulation of genes of spermatogenesis in sterile hybrid males might be independent of the sterility phenotype and instead driven by rapid interspecies male-sex regulatory divergence, leading to incompatibilities between *cis* and *trans* elements in a hybrid genome. A problem with several prior studies is that the analysis of gene expression in F1 sterile hybrids compounds both the sterility and genome divergence variables, making it hard to disentangle their respective roles.

Here we test the male-sex rapid divergence hypothesis by creating fertile hybrid backcross males, thus removing the sterility phenotype. We used a qRT-PCR gene specific rather than a genome-wide approach on the basis that genome-wide comparisons have already been conducted in comparisons of *D. simulans*, *D. mauritiana* and their sterile male progeny. Such studies have found three spermatid differentiation genes to be consistently down regulated in sterile hybrids; *don juan*, *Mst84Dc* and *Mst98Ca* [17], [19]. In a recent study where gene expression was assayed in male and female hybrids relative to parental species using a gene-specific target approach (qRT-PCR) with tissue-specific RNA sources, significant testes-specific down regulation was found for the pre-meiotic gene *bam* and the meiotic arrest gene *spermatocyte arrest* (*sa*) [26]. We chose to sample three of the previously identified five genes, that cover the three major developmental stages of spermatogenesis; premeiotic (*bam*), meiotic (*sa*) and postmeiotic (*Mst98Ca*). It is possible that down regulation of gene expression for the two other postmeiotic genes (*Mst84Dc* and *don juan*) might be linked to sterility. However, our goal was to test whether down regulation of gene expression can be caused by incompatible regulatory interactions in the absence of sterility. We measured gene expression in testes samples from thirty-five fertile *D. simulans*–*D. mauritiana* hybrid backcross strains. We found that X-chromosome *D. mauritiana* introgressions cause significantly less down regulation of our targeted spermatogenesis genes than autosomal introgressions on both the second (*trans*) and the third (*cis* and/or *trans*) chromosome. Our results show that fertile heterospecific hybrids can display similar levels of spermatogenesis gene down regulation as those detected in sterile hybrids. Such low levels of spermatogenesis genes expression can be explained by rapid evolution of divergent regulatory elements placed in heterozygosity in hybrids, in the absence of sterility.

## Materials and Methods

### Creation of Introgression (IG) Strains

**Figure 1:**
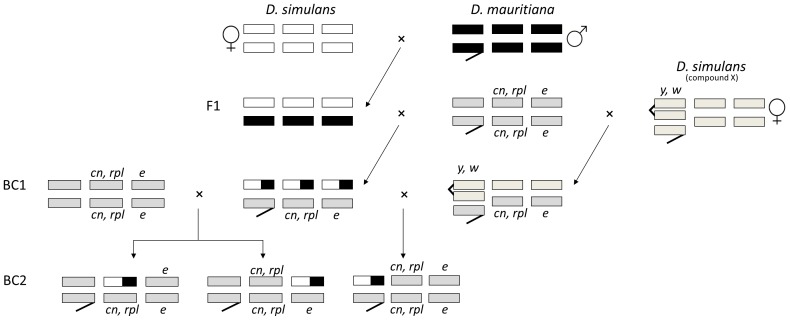
Creation of chromosome introgression (IG) strains. Black bars are used for *D. mauritiana* chromosomes and non-black for *D. simulans*.

Fly stocks were reared in cornmeal–molasses–yeast–agar (CMYA) medium at 24°C on a 12 h light–dark cycle. Virgin flies were collected and maintained in CMYA vials with no more than 20 flies per vial. F1 hybrid females from a cross between *D. simulans* (sim2 – from Dr. AG Clark) and a *D. mauritiana* stock from the Drosophila Species Stock Center (DSSC) (14021-0241.01) were backcrossed to *D. simulans* (DSSC: 14021-0251.259) homozygous for recessive markers *cinnabar* (second chromosome), *ripple* (second chromosome), and *ebony* (third chromosome). Single male offspring (BC1) from this cross were backcrossed to single *D. simulans* (DSSC: 14021-0251.259) females. Male offspring (BC2) were selected based on their phenotypes to establish second (wild type eyes, wild type wings, and ebony body colour) and third (cinnabar eyes, ripple wings, and wild type body colour) chromosome introgression strains (Fig. 1). Autosomal introgressions were maintained by selecting male progeny based on phenotype and crossing to *D. simulans* (DSSC: 14021-0251.259). For the creation of X chromosome introgressions, the same series of crosses were followed up until BC1, when single male flies were crossed to females created by the cross of *D. simulans* compound X females (stock donated by Dr. DC Presgraves) with *D. simulans* males of the phenotypic marker stock (DSSC: 14021-0251.259). BC2 males carrying an X chromosome introgression were selected based on phenotype (cinnabar eyes, ripple wings, and ebony body colour) (Fig.1). Flies with the X-introgression were maintained by selecting BC2 male progeny based on phenotype and crossing to virgin females of the *D. simulans* compound X strain.

### RNA Extraction, cDNA Synthesis, and qRT-PCR

Three biological replicates (different RNA samples) were analyzed for each IG strain and the interspecies sterile hybrids. Virgin males were aged for 4–6 days, at which point testes (with seminal vesicles) from five males were dissected and rinsed in 1× PBS. Total RNA was extracted from the dissected testes using the RNeasy plus mini kit (Qiagen) and used as the template for cDNA synthesis using poly T primers to avoid interference from other types of RNA (iScript cDNA synthesis kit, Bio-Rad). Reverse transcribed cDNA was quantified so that equal amounts of total cDNA were used for each sample.

We tested differences in gene expression using qRT-PCR for three target genes (bam, sa and Mst98Ca) after normalization relative to two reference housekeeping genes (RpL32 and RpS18). The use of two different housekeeping genes helps validate results that might be otherwise biased by single gene inconsistencies across hybrids. RpL32 has been commonly used as a normaliser in Drosophila gene expression studies [22], [26], [27] while RpS18 has been less used in Drosophila but commonly used in other insects [28], [29]. All qRT-PCR reactions were performed using the IQ SYBR GREEN quantitative real time PCR kit (Bio-Rad). QRT-PCR cycling conditions were similar for all target genes, with an original denaturing step at 95°C for 5 minutes, followed by 34 cycles of 95°C for 45 seconds, 58°C (bam and Mst98Ca) or 60°C (sa) for 30 seconds and 72°C for 45 seconds. The housekeeping genes (RpL32 and RpS18) were amplified to match the cycling conditions of the target genes. To control against genomic DNA contamination, primers were designed to span an exon/intron boundary when possible. All primers were designed using Primer3 (http://frodo.wi.mit.edu/) to amplify 58–72 bp products. The efficiency of all primer pairs was tested by creating a standard curve using the threshold cycles (CT) generated from a dilution series of a template (Table 1).

**Table 1:** Primers used in this study.

Gene	Forward	Reverse	Design	Product size	Efficiency
*bam*	gcgcagaccaatcagcaat	gatcaatgcggacaagttcca	Exon splice	58bp	113%
*sa*	tcgagggctttatgggttatca	tgccgagaaatgaggtctttg	Intron span	70bp	108%
*Mst98Ca*	ccccgtgtccgtacgagt	agtgggtgttccagggagtt	None	72bp	96%
*RpL32*	aagcggcgacgcactct	tgctaagctgtcgcacaaatg	Exon splice	63bp	101%
*RpS18*	agcgcgccggtgagt	tactgcagagggttggagatga	Intron span	70bp	101%

Primer sequences are in 5′–3′ direction.

Exon splice = At least one of the primers spans over two exons. Intron span = primers flank an intron. None = primers do not splice an exon and they do not flank an intron.

### Gene Expression Data Analysis

Gene expression was determined using the Livak method [30]. The ΔC_T(test)_ was calculated by subtracting the C_T_ of the reference gene (*RpL32* or *RpS18*) from the C_T_ of the target gene for each IG strain. The ΔC_T(calibrator)_ was calculated by subtracting the C_T_ of the reference gene (*RpL32* or *RpS18*) from the C_T_ of the target gene for the parental strain (*D. simulans* 14021-0251.259). We tested for down regulation of the test (IG strains) relative to the calibrator (*D. simulans*) by using a log_2_ transformation of 2^−ΔΔC^_T_ values, where ΔΔC_T_ is the difference in ΔC_T_ estimates between the test samples and the calibrator.

### Fecundity Assay

We measured the number of progeny produced by *D. simulans* females (fecundity) when mated to males of the different IG strains. We crossed five naïve males aged 4–6 days from each IG strain with five virgin females of the *D. simulans* strain (14021-0251.259) used to create and maintain the IG strains, as well as crossing *D. simulans* males and females. The crosses were replicated four times (four vials per treatment). Flies were dumped on the seventh day after mating and offspring were collected and counted on the fourteenth, nineteenth, and twenty-fourth days after oviposition began.

## Results and Discussion

Out of 300 strains started for the recovery of second and third chromosome introgressions only 28 (15 in the second and 13 in the third chromosome) could be established as stocks. Similarly, only 7 out of 250× chromosome introgressions were recovered. The low number of introgression strains established is not surprising given that the backcross (BC1) males used to start the stocks were, on average, heterozygous for half of their genome (Fig. 1). Thus, it is likely that most introgressions seriously impaired fertility and viability. The lower percentage of X chromosome (2.8%) than second (5%) and third (4.3%) introgression strains recovered might be explained by the higher density of male sterility factors on the X chromosome than the autosomes [31]–[33].

Expression of spermatogenesis target genes was measured using two different housekeeping genes, *RpL32* and *RpS18*, as references. Variation in expression of both housekeeping and target genes is expected due to qualitative and quantitative differences in RNA extraction and cDNA synthesis. An effective housekeeping gene should not contribute to but help correct for spurious differences across tested samples. We found significant correlation between C_T_ values of the target gene and the housekeeping reference genes for all the genes assayed (*RpL32*: R_bam_ = 0.759, P<0.001; R_sa_ = 0.833, P<0.001; R_Mst98Ca_ = 0.789, P<0.001. *RpS18*: R_bam_ = 0.726, P<0.001; R_sa_ = 0.907, P<0.001; R_Mst98Ca_ = 0.831, P<0.001). The positive correlation between effects of chromosomal introgressions on gene expression of both reference (housekeeping) and target (spermatogenesis) genes exemplifies the importance of using relative measures of expression to assert gene specific effects. To further correct for possible biases introduced by a specific housekeeping gene, we only report significant effects that are consistent after normalization of gene expression with both *RpL32* and *RpS18*.

**Figure 2:**
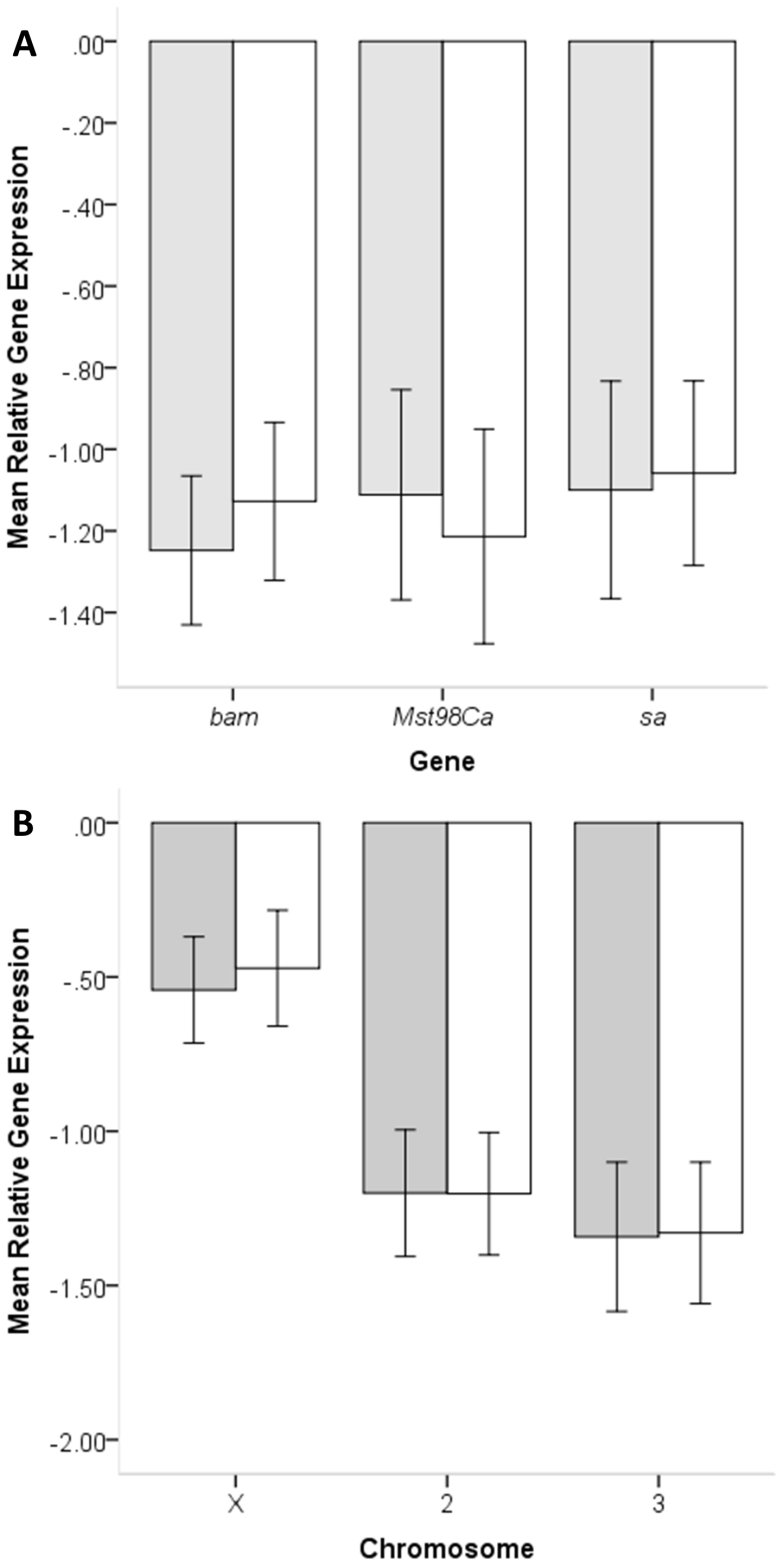
Differences in mean relative gene expression among genes (A) and chromosomes (B). Relative gene expression is shown as log_2_ deviations from *D. simulans* and using *RpL32* (grey bars) and *RpS18* (white bars) as housekeeping reference genes (see Materials and Methods). A) Mean gene expression averaged across all introgression strains and B) mean expression per site of chromosome introgression. Error bars are 95% confidence intervals.

Comparisons of gene expression revealed some level of down regulation in all introgression strains relative to *D. simulans* but there were no differences among genes tested (*RpL32*: F_2,305_ = 0.531; *P* = 0.589; *RpS18*: F_2,305_ = 0.666; *P* = 0.514) (Fig. 2A). However, there were significant differences among chromosomes (*RpL32*: F_2,305_ = 7.786; *P*<0.001; *RpS18*: F_2,305_ = 9.623; *P*<0.001) due to significantly less gene down regulation among X chromosome than autosomal introgressions (Fig. 2B). We detected no gene×chromosome interaction effects (*RpL32*: F_4,305_ = 0.582; P = 0.676; *RpS18*: F_4,305_ = 0.243; P = 0.914). Thus, the amount of gene down regulation experienced by the IG strains was similar across genes but the X chromosome had a smaller effect on target gene expression than autosomal introgressions. Several studies on the evolution of genes with male biased expression have suggested a demasculinization of the Drosophila X chromosome [34]–[39]. However, others have recently challenged the demasculinization of the X chromosome hypotheses and shown that it might be an artefact of the reduced dosage of X-linked genes [40], [41]. Our inability to find X chromosome regulatory elements affecting the expression of male (spermatogenesis) genes could be prematurely taken to support demasculinization of the X chromosome. However, we cannot fully rule out the possibility that the lesser effect of the X chromosome might be somehow biased by our own selection strategy against sterility. It is possible that, due to the larger density of *D. simulans*–*D. mauritiana* male sterility factors on the X chromosome than autosomes [32], we might have selected against regulatory elements linked to sterility, and for smaller X chromosome than autosomal introgressions. Only a large-scale genotyping experiment to compare both size and distribution of the introgressions would allow us to discern between the two possible explanations.

**Figure 3:**
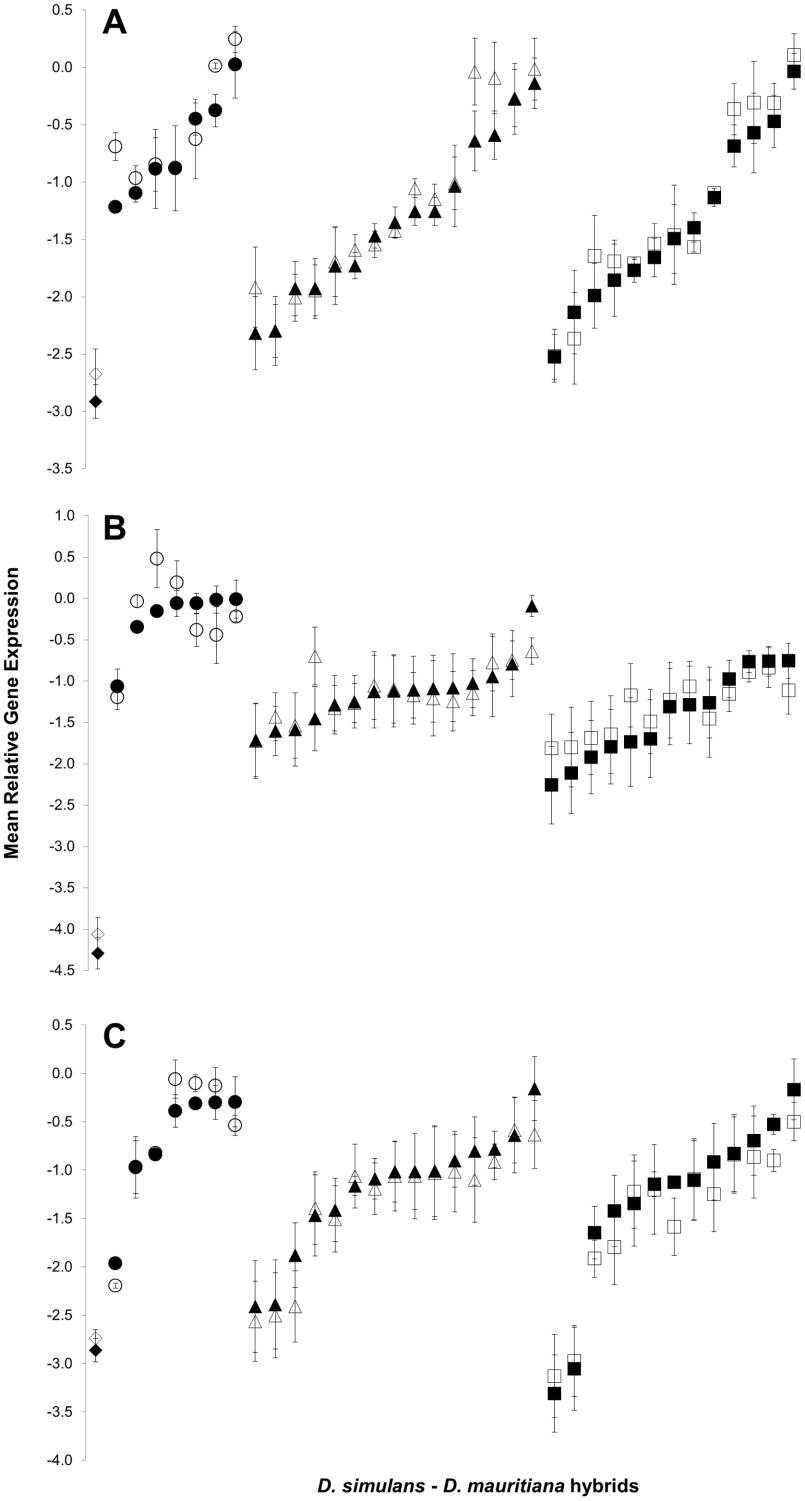
Mean relative gene expression of each introgression strain as well as sterile F1 male hybrids. Mean relative gene expression estimated as described in figure 2, and associated errors, are shown using *RpL32* (solid symbols) and *RpS18* (open symbols) as reference genes for *bam* (A), *sa* (B) and *Mst98Ca* (C). The F1 sterile male hybrid mean expression is shown as diamonds, while IG strains data are separately shown for X (circles), second (triangles) and third (squares) chromosome introgressions and arranged along the X-axis in order of decreasing severity of down regulation.

**Figure 4:**
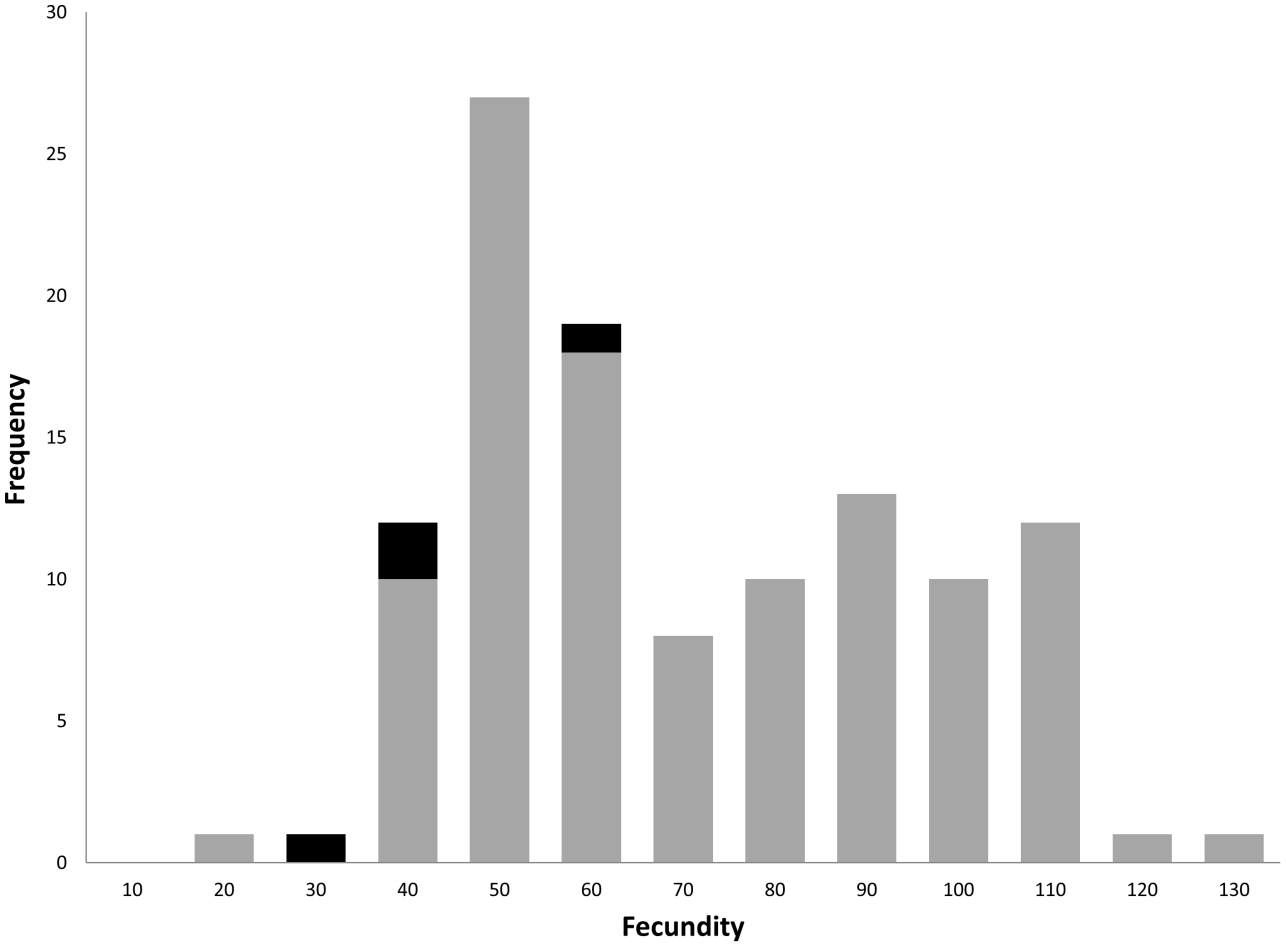
Frequency distribution of fecundity for *D. simulans* and introgression (IG) strains. Fecundity was measured as the total number of progeny produced by groups of five females (see Materials and Methods). *D. simulans* fecundity is shown as black bars.

Some autosomal IG strains show levels of average relative gene under expression (relative to *D. simulans*) similar to sterile hybrids (Fig. 3). Because gene expression could be affected by differences in fertility between IG strains, we counted number of progeny (fecundity) produced by *D. simulans* females when mated to different introgression strains or *D. simulans* males. While counting progeny produced does not directly measure fertility, the purpose of our measurement was to determine whether some IG strains were significantly less fecund than *D. simulans*. In the event of lower fecundity of IG males, a more precise measure of fertility would be needed. We found no significant differences in total fecundity between IG strains and *D. simulans* (F_28,116_ = 1.289; *P* = 0.187) (Fig. 4) and the results were consistent if we analyzed female fecundity at different intervals after oviposition began (14 days: *P* = 0.202, 19 days: *P* = 0.566 and 24 days: *P* = 0.260). There were also no significant correlations between progeny produced and gene expression for either *bam* (R = −0.021; *P* = 0.824), *sa* (R = −0.04; *P* = 0.686), or *Mst98Ca* (R = −0.138; *P* = 0.147). Our backcross approach created fertile hybrids with gene down regulation as severe as sterile hybrids. This indicates that gene under expression can be explained by gene misregulation in a hybrid genome.

Finally, our results show that down regulation of spermatogenesis gene expression could be attributed to incompatible regulatory interactions between the genomes of two species in the absence of sterility. However, the specific regulatory interactions in our IG fertile strains are not identical to those in an F1 interspecies sterile hybrid. One major difference is that IG strains are only hybrids for parts of a single chromosome. Thus, we have not sampled all possible incompatible interactions. However, the fact that we were able to see severe gene down regulation in IG strains by sampling only some of all possible interspecies regulatory interactions attests to the fact that regulatory interspecies incompatibilities can cause spermatogenesis gene down regulation. The other major difference is that the introgressed chromosome in IG strains is a *D. simulans* (sim2, California) recombinant chromosome in a different *D. simulans* strain (sim259, Mexico) background. It is then possible that differences in gene regulation between the two different strains of *D. simulans* could cause or be a major contributor to the observed down regulation of the target genes. Although we have not tested such possibility here, we find it extremely unlikely based on prior data showing that, at least for *bam* and *sa*, fertile male hybrids between an African and a North American (sim2) strain of *D. simulans* showed no significant differences in gene expression when compared to their parents [26]. This result is particularly telling given that *D. simulans* African populations have greater genome polymorphism than North American populations and that genetic variation in the New World is a subset of that found in Africa [42].

## Conclusions

Our study specifically targeted three spermatogenesis genes on the basis of results from previous genome scan and gene-specific studies that have shown them to be significantly down regulated in testes/sperm of sterile hybrids. Our results do not rule out the possibility of a link between sterility and gene misexpression, in fact a prior study carefully assayed and properly established a link between under expression of a sperm motility gene (*Acyp*) and sterility [21]. Instead, our study shows that heterospecific fertile introgressions can show levels of gene down regulation as severe as sterile hybrids. Single gene down regulation in sterile individuals, from either genome or gene-specific assays, should not be taken as causation for sterility. The significant effect of fertile heterospecific introgressions on the expression of both premeiotic and postmeiotic genes lends support to the hypothesis that rapid evolution of divergent regulatory elements placed in heterozygosity in hybrids is able to effect gene expression in the absence of sterility.
